# Prediction of adenomyosis according to revised definitions of morphological uterus sonographic assessment features

**DOI:** 10.3389/fmed.2024.1387515

**Published:** 2024-08-08

**Authors:** Onur Yavuz, Asli Akdöner, Mehmet Eyüphan Özgozen, Begüm Ertan, Sefa Kurt, Emine Cagnur Ulukuş, Mehmet Güney

**Affiliations:** ^1^Department of Obstetrics and Gynecology, Dokuz Eylül University School of Medicine, Izmir, Türkiye; ^2^Department of Pathology, Dokuz Eylül University School of Medicine, Izmir, Türkiye

**Keywords:** adenomyosis, direct feature, indirect feature, 3D transvaginal ultrasonography, 2D transvaginal ultrasonography

## Abstract

**Objectives:**

This study aimed to predict the diagnosis of adenomyosis by revised definitions of morphological uterus sonographic assessment (MUSA) features in individuals who had hysterectomy.

**Methods:**

This was retrospective cohort research conducted at a tertiary facility. Between January 2022 and January 2023, 196 individuals who had hysterectomy were analyzed in the research. The revised definitions of MUSA features of the adenomyosis approach were used to record the direct and indirect results of the sonography. The cases were classified as Group 1 (adenomyosis; *n* = 40, 20.4%) and Group 2 (control; *n* = 156, 79.6%) according to histopathology reports.

**Results:**

Hyperechogenic islands and echogenic subendometrial buds and lines were the most predictive direct features (*p* = 0.02). Globular uterus and irregular junctional zone were the most predictive indirect features (*p* = 0.04; *p* = 0.03, respectively). Among all indirect features, the globular uterus was the most predictive (*p* = 0.02). Total feature >4 was determined as the significant cutoff value to predict adenomyosis (*p* < 0.001).

**Conclusion:**

This study shows that combinations with a total number of features >4 can be practically used in the evaluation of adenomyosis using the revised definitions of MUSA features.

## 1 Introduction

The benign uterine condition known as adenomyosis is identified by the existence of stroma and endometrial glands in the myometrium (1). Whether or not there is a hypertrophic myometrium nearby, it may appear as a localized or widespread lesion in the inner or outer myometrium (2). The overall prevalence of histopathologically confirmed adenomyosis was reported as between 20.9 and 36.4% (3, 4). It is also stated that adenomyosis peaks between the ages of 40 and 59 (3).

While the diagnosis of adenomyosis is definitively made histopathologically, it can only be predicted by non-invasive imaging methods. Nowadays, transvaginal ultrasonography (TVS) is the first-line imaging technique in the diagnosis of adenomyosis (5). In a meta-analysis in which the diagnosis of adenomyosis was confirmed histopathologically, the sensitivity and specificity of preoperative TVS for predicting adenomyosis were found to be both 78% (6). It has been noted that three-dimensional (3D) TVS increases the accuracy of adenomyosis diagnosis (6).

Diagnostic sonographic characteristics of adenomyosis were examined in many studies in the literature (4, 7, 8). Van Den Bosch et al. reported MUSA features to optimize and standardize sonographic markers of adenomyosis (9). After that, MUSA features were revised and updated to define adenomyosis by a modified Delphi procedure study (5). Everyone agreed that the ultrasonographic signs of adenomyosis in the MUSA features should be classified as either indirect (asymmetrical myometrial thickening, globular uterus, fan-shaped shadowing, translesional vascularity, inconsistent junctional region, and interrupted junctional region) or direct (myometrial cysts, hyperechogenic islands, echogenic subendometrial buds, and lines) (5). The most recent study on this subject, revised definitions of MUSA features of adenomyosis, highlighted a gap in the literature as further investigation of the accuracy of the existence of one or more indirect and/or direct features to diagnose adenomyosis (5).

Based on this perspective, we aimed to predict the diagnosis of histopathologically confirmed adenomyosis by utilizing the revised and updated MUSA ultrasonographic features (one or more indirect and/or direct features) in patients who underwent hysterectomy.

## 2 Materials and methods

This was retrospective cohort research carried out at a tertiary center. Informed consent was obtained from all participants in this research. The research was performed in compliance with the principles of the Helsinki Declaration. Institutional ethics committee approval was provided (File number: 7737-GOA, Registration number: 2023/02-13). Between January 2022 and January 2023, 214 patients who underwent hysterectomy were included in the research. Individuals with indications of cervical, uterine, and adnexal malignancy were excluded from the study (*n* = 18).

Data from 196 patients were analyzed.

During the preoperative period within the last week, the patients were examined with 3D TVS (General Electric^®^ Voluson E8 with a 4–9 MHz 3D transvaginal probe). Sonographic evaluations were performed by three gynecologists working at our institution whose special interest is in endometriosis and adenomyosis ultrasonography. The sonographic examinations were performed by two gynecologists, 30 and 10 years old (MG and OY, respectively). In cases of discrepancies, a third gynecologist with 6 years of experience (MEÖ) ensured consensus. The presence of leiomyoma (location, site, number, and maximum diameter) was recorded. The location of leiomyoma was defined as the anterior and posterior sides of the uterus. The site of leiomyoma was classified as type 0–7, in accordance with the current literature that defines the classification of leiomyoma (10). If more than one leiomyoma was detected, the characteristics of the largest myoma were used as the basis. To predict adenomyosis, the revised definitions of MUSA features, including direct (myometrial cysts, hyperechogenic islands, echogenic subendometrial buds, and lines), indirect (asymmetrical myometrial thickening, globular uterus, fan-shaped shadowing, translesional vascularity, irregular junctional zone, and interrupted junctional zone), and the total number of signs (direct + indirect), were accepted as a reference, and the findings were documented (5). The demographic characteristics, clinical findings, surgery indications, and surgery type of the patients were recorded. Following the surgeries, the pathology materials were analyzed by a single experienced gynecopathologist (EÇU). Macroscopically, an enlarged uterus, a spherical and/or asymmetrical uterus, and a thick, irregularly fasciculated myometrium with tiny gaps were used to diagnose adenomyosis. When an adenomyoma resembles an intramural myoma or when the adenomyotic lesions are limited to the uterine wall, it is referred to as focal adenomyosis (11). Histologically, the existence of ectopic endometrial glands and/or stroma linked to neighboring smooth muscle hypertrophy and hyperplasia located 2.5 mm past the endometrial–myometrial interface when seen via a low-power microscope established the diagnosis of adenomyosis (11). The histopathological diagnosis of endometrioma was reported in patients who underwent unilateral or bilateral salpingo-oophorectomy along with hysterectomy. The characteristics of the leiomyomas assessed preoperatively were confirmed histopathologically. The cases were classified as Group 1 (adenomyosis; *n* = 40, 20.4%) and Group 2 (control; *n* = 156, 79.6%) according to histopathology reports.

Analyses were performed with SPSS version 26.0 (IBM Inc., Chicago, IL, USA). Normality analysis was performed according to the Kolmogorov–Smirnov test. Not normally distributed variables were analyzed with the Mann–Whitney *U*-test. These results were expressed as median (minimum–maximum) values for each group. The chi-square test and Fisher's precision test were used in the analysis of categorical data. These were presented as counts and percentages (%). An inter-rater reliability analysis was performed for direct and indirect ultrasonography findings. For this purpose, Cohen's Kappa was calculated and categorized as follows: *k* = 0–0.20, slight agreement; *k* = 0.21–0.40, fair agreement; *k* = 0.41–0.60, moderate agreement; *k* = 0.61–0.80, substantial agreement; and *k* = 0.81–1.00, almost perfect agreement. Logistic regression models were used to analyze features that may be effective in predicting adenomyosis. Receiver operating characteristic (ROC) analysis was performed to determine the area under the curve (AUC), which indicates the average sensitivity of features. The appropriate cutoff value, indicating the sum of the highest sensitivity and specificity, was calculated for the most predictive feature. The results were a 95% confidence interval (CI). The *p*-value considered statistically significant was <0.05.

## 3 Results

Demographic characteristics and clinical findings of groups are listed in Table 1. The groups were similar with regard to age, gravity, parity, body mass index (BMI), menarche age, menopausal status, and smoking habit. There was no significant difference between the groups in terms of the history of myomectomy surgery, cesarean section, curettage, oral progesterone treatment, or levonorgestrel intrauterine device treatment. Although the history of dyspareunia and chronic pelvic pain was detected at a higher rate in the adenomyosis group, none of the clinical symptoms showed statistically significant differences between the groups.

**Table 1:** Demographic characteristics and clinical findings of groups.

**Variables**	**All patients *n* = 196 (100%)**	**Group 1 (adenomyosis) *n* = 40 (20.4%)**	**Group 2 (control) *n* = 156 (79.6%)**	***p*-value**
Age (years)	49 (34–80)	48 (35–75)	49 (34–80)	0.1
Gravidy	2 (0–13)	3 (0–7)	2 (0–13)	0.4
Parity	2 (0–11)	2 (0–7)	2 (0–11)	0.4
Body mass index (%)	28 (18–43.1)	28 (20–40)	28 (18–43.1)	0.9
Menarche age (years)	13 (11–17)	13 (11–17)	13 (11–17)	0.7
Menopausal status				0.2
Premenopausal	56.1% (110/196)	65% (26/40)	53.8% (84/156)	
Postmenopausal	43.9% (86/196)	35% (14/40)	46.2% (72/156)	
Smoking habit (10/day)	40.3% (79/196)	42.5% (17/40)	39.7% (62/156)	0.8
Myomectomy surgery	40.8% (80/196)	42.5% (17/40)	40.4% (64/156)	0.8
Cesarean section	37.2% (73/196)	35% (14/40)	37.8% (59/156)	0.8
Curettage history	48% (94/156)	52.5% (21/40)	46.8% (73/156)	0.5
Oral progesterone treatment	27% (53/196)	25% (10/40)	27.6% (43/156)	0.8
Levonorgestrel intrauterine device treatment	15.3% (30/196)	22.5% (9/40)	13.5% (21/156)	0.2
Dysmenorrhea	43.9% (86/196)	35% (14/40)	46.2% (72/156)	0.2
Dyspareunia	28.6% (56/196)	35% (14/40)	26.9% (42/156)	0.3
Menometrorrhagia	40.8% (80/196)	37.5% (15/40)	41.7% (65/156)	0.7
Chronic pelvic pain	28.1% (55/196)	37.5% (15/40)	25.6% (40/156)	0.1

Ultrasound findings of the groups are listed in Table 2. Among the indirect features, the globular uterus and irregular junctional zone were observed to be significantly greater in the adenomyosis group (57.5 vs. 39.7%; *p* = 0.04, 32.5 vs. 17.3%; *p* = 0.03, respectively). Other indirect features did not differ between groups. Although myometrial cysts, which are direct features, were detected at a higher rate in the adenomyosis group, the difference was not significant. Hyperechogenic islands and echogenic subendometrial buds and line features were significantly higher in the adenomyosis group (17.5 vs. 5.8%; *p* = 0.01, 15 vs. 4.5%; *p* = 0.01, respectively). The median value of the total number of direct, indirect, and total features was significantly higher in the adenomyosis group (*p* = 0.04; *p* = 0.04; *p* < 0.01). The groups were similar in terms of leiomyoma presence, site, number, and maximum diameter. The anterior location of maximum diameter myoma uteri was higher in the control group (*p* = 0.03)

**Table 2:** Ultrasound findings of the groups.

**Variables**	**All patients *n* = 196 (100%)**	**Group 1 (adenomyosis) *n* = 40 (20.4%)**	**Group 2 (control) *n* = 156 (79.6%)**	***p*-value**
**Indirect signs**				
Asymmetrical myometrial thickening	42.9% (84/196)	55% (22/40)	39.7% (62/156)	0.08
Globular uterus	43.4% (85/196)	57.5% (23/40)	39.7% (62/156)	0.04
Fan-shaped shadowing	41.8% (82/196)	55% (22/40)	38.5% (60/156)	0.05
Translesional vascularity	5.6% (11/196)	7.5% (3/40)	5.1% (8/156)	0.5
Irregular junctional zone	20.4% (40/196)	32.5% (13/40)	17.3% (27/156)	0.03
Interrupted junctional zone	21.4% (42/196)	30% (12/40)	19.2% (30/156)	0.1
**Direct signs**				
Myometrial cysts	15.8% (31/196)	17.5% (7/40)	15.4% (24/156)	0.7
Hyperechogenic islands	8.2% (16/196)	17.5% (7/40)	5.8% (9/156)	0.01
Echogenic subendometrial buds and lines	6.6% (13/196)	15% (6/40)	4.5% (7/156)	0.01
Total number of indirect features	2 (0–7)	3 (0–7)	2 (0–6)	0.04
Total number of direct features	0.3 (0–3)	0 (0–3)	0 (0–2)	0.04
Total number of features	2 (0–9)	3 (0–9)	2 (0–7)	<0.01
Myoma uteri	61.2% (120/196)	62.5% (25/40)	60.9% (95/156)	0.8
**Location of maximum diameter myoma uteri**				0.03
Anterior wall	82.5% (99/120)	68% (17/25)	86.3% (82/95)	
Posterior wall	17.5% (21/120)	32% (8/25)	13.7% (13/95)	
**Site of maximum diameter myoma uteri**				0.7
Type 0–2	65.8% (79/120)	60% (15/25)	67.4% (64/95)	
Type 3–6	25% (30/120)	28% (7/25)	24.2% (23/95)	
Type 7	9.2% (11/120)	12% (3/25)	8.4% (8/95)	
Number of myoma uteri (cm)	3 (1–10)	3 (1–10)	3 (1–10)	0.9
Maximum diameter of myoma uteri (cm)	4 (1–10)	4 (1–10)	4 (1–10)	0.9

Interobserver agreement of ultrasound findings is shown in Table 3. Cohen's Kappa showed that there was an almost perfect agreement for asymmetrical myometrial thickening and globular uterus. Moderate agreement was found for fan-shaped shadowing and hyperechogenic islands; fair agreement was found for translesional vascularity, interrupted junctional zone, myometrial cysts, and echogenic subendometrial buds and lines; a slight agreement was found for the irregular junctional zone between observers.

**Table 3:** Interobserver agreement of ultrasound findings.

**Variables**	**Interobserver agreement rate (%)**	**Kappa (CI 95%)**	***p*-value**
**Indirect signs**			
Asymmetrical myometrial thickening	91.8%	0.83 (0.75–0.91)	<0.001
Globular uterus	92.3%	0.84 (0.77–0.92)	<0.001
Fan-shaped shadowing	70.3%	0.41 (0.29–0.53)	<0.001
Translesional vascularity	85.7%	0.31 (0.21–0.50)	<0.001
Irregular junctional zone	65.8%	0.2 (0.07–0.36)	<0.01
Interrupted junctional zone	70.9%	0.33 (0.19–0.46)	<0.001
**Direct signs**			
Myometrial cysts	87.7%	0.58 (0.43–0.73)	<0.001
Hyperechogenic islands	94.3%	0.65 (0.46–0.84)	<0.001
Echogenic subendometrial buds and lines	94%	0.59 (0.37–0.81)	<0.001

The operation and pathological outcomes of the groups are listed in Table 4. The groups were similar in terms of indication and type of surgery. Although the coexistence of myoma uteri and endometrioma was detected at a higher rate in the control group, these variables were not different between the groups. No adenomyoma coexistence was detected in the control group. This rate was reported as 15% in the adenomyosis group (*p* < 0.001).

**Table 4:** Operation and pathological findings of the groups.

**Variables**	**All patients *n* = 196 (100%)**	**Group 1 (adenomyosis) *n* = 40 (20.4%)**	**Group 2 (control) *n* = 156 (79.6%)**	***p*-value**
**Indication**				0.1
Myoma uteri	40.8% (80/196)	37.5% (15/40)	41.7% (65/156)	
Premenopausal abnormal uterine bleeding	23% (45/196)	32.5% (13/40)	20.5% (32/156)	
Uterine prolapse	18.9% (37/196)	10% (4/40)	21.2% (33/156)	
Chronic pelvic pain	6.6% (13/196)	10% (4/40)	5.8% (9/156)	
Benign adnexal mass	4.6% (9/196)	0% (0/40)	5.8% (9/156)	
Postmenopausal bleeding	6.1% (12/196)	10% (4/40)	5.1% (8/156)	
**Surgery type**				0.3
TAH + BS	3.1% (6/196)	5% (2/40)	2.6% (4/156)	
TAH + USO	1% (2/196)	0% (0/40)	1.3% (2/156)	
TAH + BSO	17.5% (35/196)	20% (8/40)	17.3% (27/156)	
TLH + BS	20.4% (40/196)	17.5% (7/40)	21.2% (33/156)	
TLH + USO	1.5% (3/196)	5% (2/40)	0.6% (1/156)	
TLH + BSO	54.1% (106/196)	52.5% (21/40)	54.5% (85/156)	
VH + BSO	2% (4/196)	0% (0/40)	2.6% (4/156)	
Coexistence of myoma uteri	61.2% (120/196)	62.5% (25/40)	60.9% (95/156)	0.8
Coexistence of endometrioma	7.9% (12/151)	15.6% (5/32)	5.9% (7/119)	0.07
Coexistence of adenomyoma	3.1% (6/196)	15% (6/40)	0% (0/156)	<0.001

TAH, total abdominal hysterectomy; TLH, total laparoscopic hysterectomy; VH, vaginal hysterectomy; BS, bilateral salpingectomy; USO, unilateral bilateral salpingo-oophorectomy; BSO, bilateral salpingo-oophorectomy.

**Figure 1:**
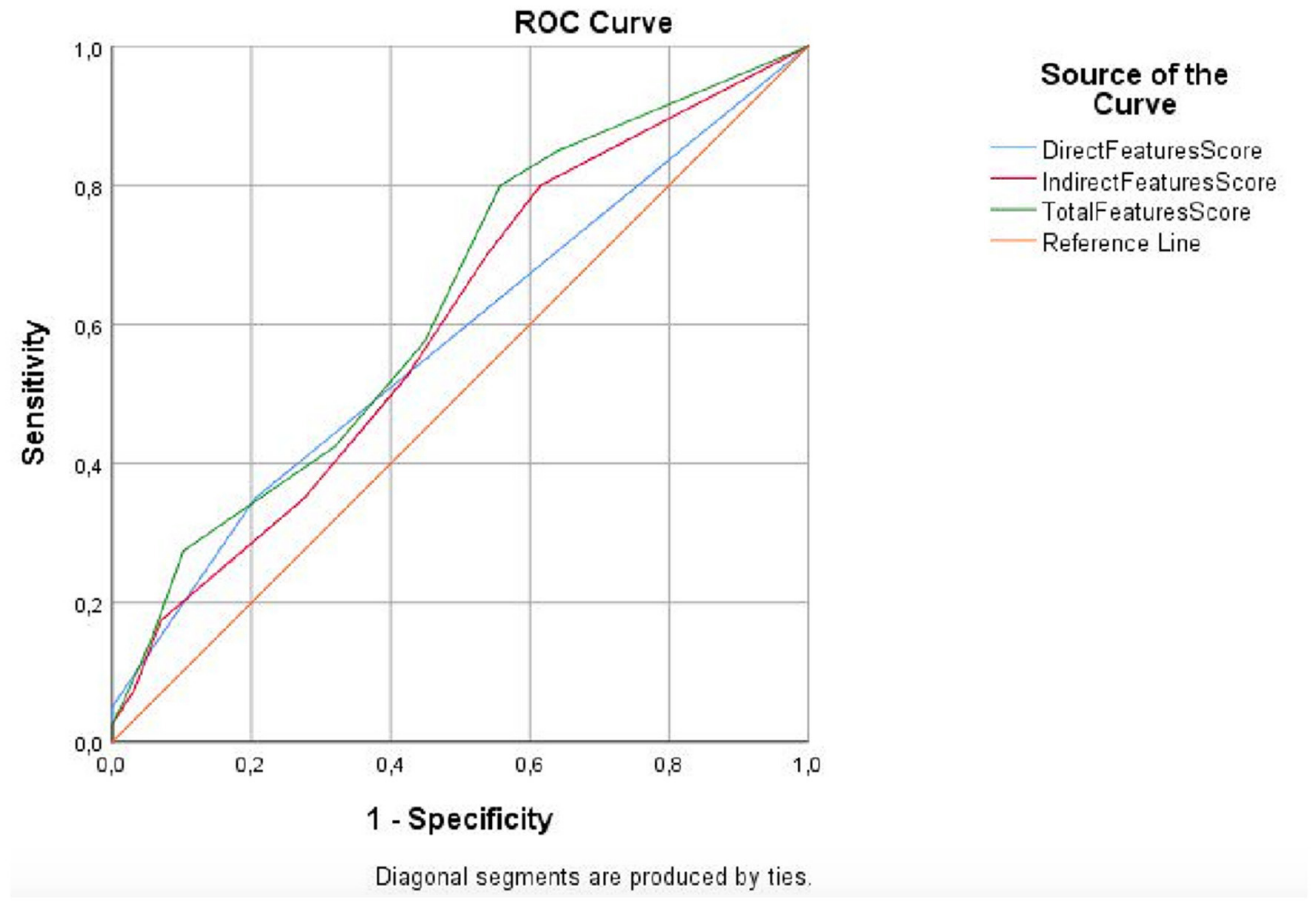
ROC curve analysis for direct, indirect, and total feature scores.

ROC analysis was conducted to calculate the cutoff score of the number of diagnostic direct, indirect, and total features for adenomyosis (Figure 1). Direct feature >1 was determined as the cutoff value to predict adenomyosis (*p* = 0.1). The AUC for the direct feature was 0.578 (95% CI, 0.473–0.620). Indirect feature >4 was determined as the cutoff value to predict adenomyosis (*p* = 0.05). The AUC for the indirect feature was 0.599 (95% CI, 0.503–0.695). Total feature >4 was determined as the cutoff value to predict adenomyosis (*p* < 0.001). The AUC for the total feature was 0.631 (95% CI, 0.536–0.725).

**Figure 2:**
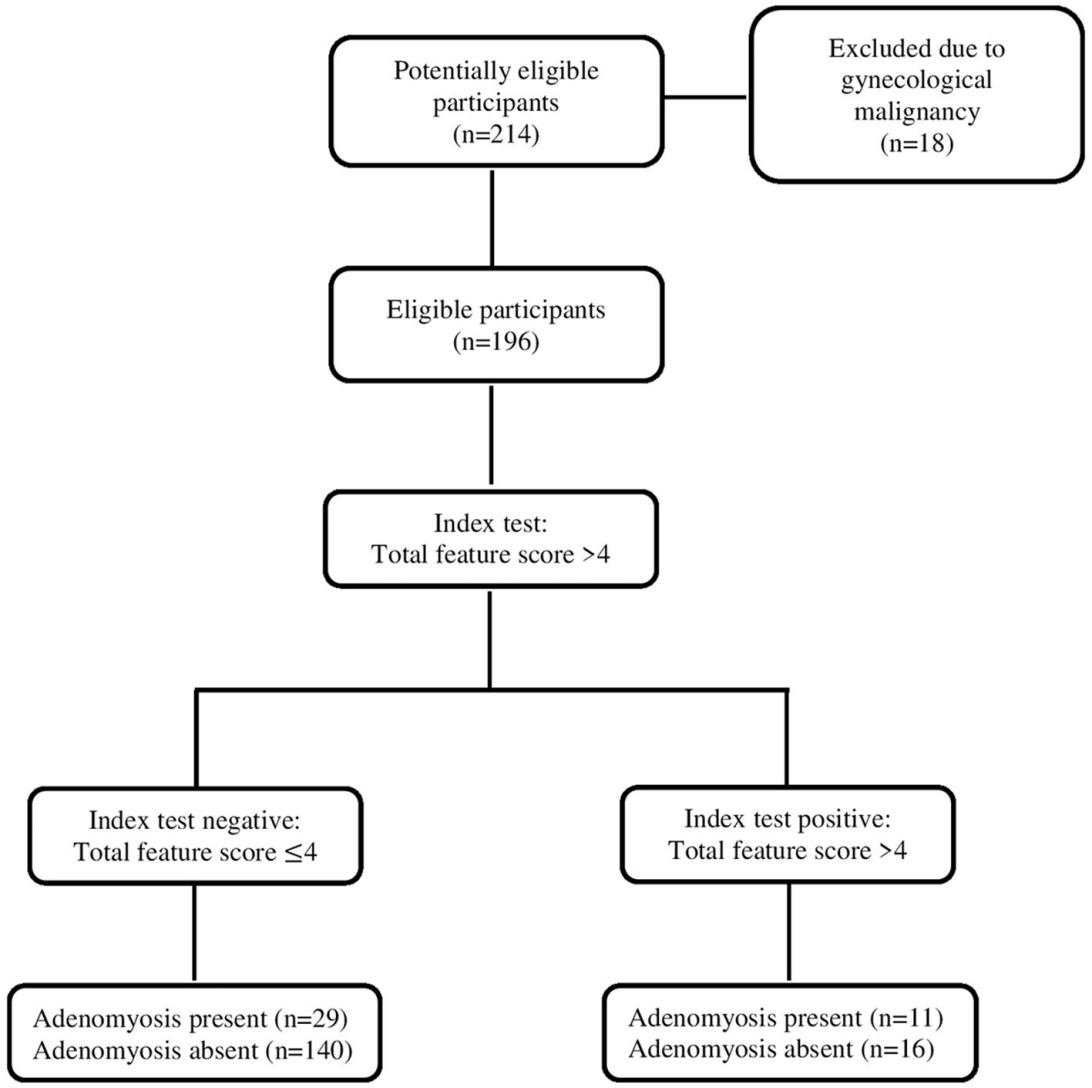
STARD diagram to report the flow of participants throughout the study.

The evaluation of likelihood ratio (LR), negative predictive value (NPV), positive predictive value (PPV), specificity, sensitivity, and accuracy of ultrasonography findings are reported in Table 5. Hyperechogenic islands had 17.5% sensitivity, 94% specificity, and 78% accuracy. However, echogenic subendometrial buds and lines had more specificity (95%) and higher accuracy (79%). Among the indirect findings, the most sensitive feature was the globular uterus (57.7%) and the most specific feature was translesional vascularity (94.8%). In addition, translesional vascularity had the highest accuracy (77%). Total feature score >4 had 27.5% sensitivity, 90% specificity, and 77% accuracy (Figure 2). There were four situations (“3 direct + ≥2 indirect”, “2 direct + ≥3 indirect”, “1 direct + ≥ 4 indirect”, and “0 direct + ≥ 5 indirect”) that met the total feature score >4 criterion. “3 direct + ≥ 2 indirect” combination had the highest specificity (100%) and accuracy (80.6%). “1 direct + ≥ 4 indirect” and “0 direct + ≥ 5 combinations” had highest sensitivity (10%).

**Table 5:** Evaluation of sensitivity, specificity, negative predictive value, positive predictive value, likelihood ratio, and accuracy of ultrasound findings.

**Variables**	**Sensitivity (%)**	**Specificity (%)**	**PPV (%)**	**NPV (%)**	**LR +**	**LR –**	**Accuracy (%)**
**Direct features**							
Myometrial cysts	17.5	79	22.5	80	0.8	1	70
Hyperechogenic islands	17.5	94	43	94	2.9	0.8	78
Echogenic subendometrial buds and lines	15	95.5	46	81	3.3	0.8	79
**Indirect features**							
Asymmetrical myometrial thickening	55	60	26.1	83.9	1.3	0.7	59
Globular uterus	57.7	60.2	27	84.6	1.4	0.7	59
Fan-shaped shadowing	55	61	26.8	84.2	1.4	0.7	60
Translesional vascularity	7.5	94.8	27.2	80	0.3	0.9	77
Irregular junctional zone	32.5	82.6	32.5	82.6	1.8	0.8	72
Interrupted junctional zone	30	80	28.5	81.8	1.5	0.8	70
Total feature score > 4	27.5	90	40	82	2.6	0.8	77
Direct feature score > 1	12.5	95	38.4	80.8	2.4	0.9	78
Indirect feature score > 4	17.5	93	38.8	81.4	2.4	0.8	77.5
0 direct + ≥5 indirect	10	95.5	36.3	80.5	2.2	1	78
1 direct + ≥4 indirect	10	95.5	22	79.6	2.2	1	77
2 direct + 0 indirect	NA	98.7	NA	79.3	NA	0.9	78.5
2 direct + 1 indirect	NA	98	NA	79.3	NA	0.9	78
2 direct + 2 indirect	NA	99.3	NA	79.4	NA	0.9	79
2 direct + ≥3 indirect	NA	98.7	60	80	5.7	1	80.1
3 direct + 0 indirect	NA	100	NA	79.5	NA	1	79.5
3 direct + 1 indirect	NA	100	NA	79.5	NA	1	79.5
3 direct + ≥2 indirect	5	100	100	95.1	NA	1	80.6

PPV, positive predictive value; NPV, negative predictive value; LR, likelihood ratio; NA, not acceptable.

Tables 6, 7 included logistic regression analysis for predicting adenomyosis. While hyperechogenic islands and echogenic subendometrial buds and lines were significant in univariate logistic regression analysis (*p* = 0.02), they were not in multivariable analysis (*p* > 0.5). In univariate logistic regression analysis, the globular uterus and irregular junctional zone were significant (*p* = 0.04; *p* = 0.03, respectively). In multivariate logistic regression analysis, the globular uterus was the only feature that showed a significant difference (*p* = 0.02).

**Table 6:** Results of univariate logistic regression analysis for predicting adenomyosis.

**Variables**	**B**	**S.E**.	**Wald**	**OR (95% CI)**	***p*-value**
Myometrial cysts	0.154	0.472	0.107	1.167 (0.463–2.940)	0.7
Hyperechogenic islands	−1.243	0.540	5.305	0.289 (0.100–0.831)	0.02
Echogenic subendometrial buds and lines	−1.323	0.588	5.067	0.266 (0.084–0.843)	0.02
Asymmetrical myometrial thickening	−0.617	0.357	2.978	0.540 (0.268–1.087)	0.08
Globular uterus	−0.718	0.359	3.999	0.488 (0.241–0.986)	0.04
Fan-shaped shadowing	−0.671	0.358	3.512	0.511 (0.254–1.031)	0.06
Translesional vascularity	−0.405	0.702	0.334	0.667 (0.169–2.637)	0.5
Irregular junctional zone	−0.833	0.389	4.372	0.435 (0.199–0.949)	0.03
Interrupted junctional zone	−0.588	0.400	2.155	0.556 (0.253–1.218)	0.1

CI, confidence interval; OR, odds ratio.

**Table 7:** Results of multivariable logistic regression analysis for predicting adenomyosis.

**Variables**	B^*^	S.E.^*^	**B^†^**	**T^†^**	**(95% CI)**	***R***	***R* ^2^**	***p*-value**
Consant	0.599	0.296	–	2.025	0.015–1.182	0.08	0.08	0.04
Hyperechogenic islands	0.189	0.106	0.126	1.789	−0.019 to 0.397	–	–	0.07
Echogenic subendometrial buds and lines	0.226	0.116	0.139	1.954	−0.002 to 0.454	–	–	0.05
Globular uterus	0.127	0.056	0.157	2.255	0.16 to 0.239	–	–	0.02
Irregular junctional zone	0.110	0.071	0.110	1.566	−0.029 to 0.250	–	–	0.1

^*^Non-standard coefficients.

^†^Standard coefficients.

CI, confidence interval.

## 4 Discussion

In this current study, we aimed to predict the diagnosis of histopathologically confirmed adenomyosis in patients undergoing hysterectomy using the revised definitions of MUSA features. The overall prevalence of adenomyosis was 20.4%. Hyperechogenic islands and echogenic subendometrial buds and lines were the most predictive direct features. The globular uterus and irregular junctional zone were the most predictive indirect features. Among all indirect features, the globular uterus was the most predictive. Total feature >4 was determined as the statistically significant cutoff value to predict adenomyosis.

The accuracy of TVS criteria in the adenomyosis diagnosis was investigated by Kepkep et al. (8). The sensitivity, specificity, and accuracy of TVS in the diagnosis of adenomyosis were 80.8, 61.4, and 68.6%, respectively (8). In another study, Bazot et al. reported the sensitivity (80.9%), specificity (100%), and accuracy (82.6%) of TVS for the diagnosis of adenomyosis in individuals with menometrorrhagia (4). Unfortunately, the sensitivity of TVS was found to be poor (38.4%) in an unselected patient population scheduled for hysterectomy (4). Unlike these studies, our criteria were defined according to the revised definitions of MUSA features of adenomyosis (5).

Naftalin et al. reported the histopathological coexistence of leiomyoma and adenomyosis as 21%, and the presence of leiomyoma without adenomyosis as 20% (3). In our study, the coexistence of adenomyosis and leiomyoma was three times higher than that reported in the literature. This rate was greater than the control group, but it was not statistically significant. Although it was thought that including patients with various site, number, and maximum diameter leiomyomas in our study group would affect sonographic sensitivity and specificity, there was no difference between the groups. Only, the rate of anterior location of the maximum diameter myoma uteri was statistically higher in the control group. Exacoustos et al. reported that the accuracy of the overall two-dimensional (2D) and three-dimensional (3D TVS) diagnoses, depending on whether two or more of the particular ultrasonographic characteristics were present, was 83 and 89%, respectively (7). There was no significant change in the specificity and accuracy of 3D sonography parameters compared to 2D sonography parameters, although there was a significantly increased sensitivity and NPV in the diagnosis of adenomyosis (7). Despite the presence of leiomyomas with various characteristics in our study, the fact that sonography evaluation was performed with 3D TVS enables better determination of the sonographic features as stated in the literature (5, 7).

A three-round modified Delphi procedure was designed among gynecologists with expertise in the ultrasonographic diagnosis of adenomyosis to reach a consensus. The Delphi procedure is a qualitative research method aimed at determining the collective opinions of experts on a specific subject. Two rounds of surveys were conducted. The surveys included ultrasound images and video clips of the uteri of women suspected to have adenomyosis. The purposes of presenting the images and video clips were: (1) to investigate the agreement among experts regarding the presence of MUSA features that may necessitate a revised definition due to poor agreement; (2) to gather suggestions regarding revised definitions; and (3) to reach a consensus on the proposed revised definitions. In the revised definitions of MUSA features of adenomyosis, consensus was achieved regarding the categorization of MUSA features into direct and indirect ultrasound indicators of adenomyosis (5). Direct features signify the existence of ectopic endometrial tissue within the myometrium (5). The consensus was attained at rates of 80, 93.3, and 60% for hyperechogenic islands, myometrial cysts, and echogenic subendometrial buds and lines, respectively (5). In our study, the interobserver agreement rate for hyperechogenic islands, myometrial cysts, and echogenic subendometrial buds and lines was found to be 94.3, 87.7, and 94%, respectively. There was a fair agreement for myometrial cysts and echogenic subendometrial buds and lines, while a moderate agreement was found for hyperechogenic islands. Indirect features encompass those that arise as secondary effects of the existence of endometrial tissue in the myometrium, including muscular hypertrophy (resulting in a globular uterus) or artifacts (e.g., shadowing). Consensus was attained at rates of 86.7, 86.7, 100, 80, 66.7, and 60% for the globular uterus, asymmetrical myometrial thickening, fan-shaped shadowing, translesional vascularity, irregular junctional zone, and interrupted junctional zone, respectively (5). In our study, total agreement for globular uterus, asymmetrical myometrial thickening, fan-shaped shadowing, translesional vascularity, irregular junctional zone, and interrupted junctional zone was found to be 92.3, 91.8, 70.3, 85.7, 65.8, and 85.7%, respectively. Asymmetric thickening was defined as the thickness difference between the anterior and posterior myometrial walls exceeding 5 mm or the ratio between the anterior and posterior wall thickness being well-above 1 or well-below 1 (5). A globular uterus was defined as one in which the myometrial serosa deviates from the cervix in at least two directions, rather than following a path parallel to the endometrium, and the measured diameters of the uterine corpus are approximately equal. We based our study on the suggested criteria (5). In our study, compared with the Delphi study, the total interobserver agreement for asymmetric myometrial thickening and globular uterus was higher, with almost perfect interobserver agreement.

Our results revealed higher total agreement for echogenic subendometrial buds and lines as well as the interrupted junctional zone in comparison to the modified Delphi study. Conversely, fan-shaped shadowing exhibited a lower total agreement in our study. We posit that these discrepancies may be attributed to factors such as the presence of myoma uteri, the number of myoma uteri, the site of the maximum diameter of myoma uteri, the location of myoma uteri, and the overall dimensions of myoma uteri.

In our study, myometrial cysts of all sizes were included based on consensus among experts in the revised definitions of MUSA features of adenomyosis. Myometrial cysts were detected in 15.4% of the patients in the adenomyosis group. The sensitivity, specificity, and accuracy of myometrial cysts were 17.5, 79, and 70%, respectively. Bazot et al. reported that the presence of a myometrial cyst on TVS had low sensitivity (65.3%) but high specificity (97.5%) for adenomyosis, regardless of the patient group (4). According to Exacoustos et al., the existence of a myometrial cyst as the sole diagnostic feature for adenomyosis was detected in 53% of patients, with a high specificity (98%) and the highest accuracy (78%) (7). In contrast, myometrial heterogeneity alone emerged as the most sensitive feature (88%) (7). Kepkep et al. demonstrated that myometrial heterogeneity was the most sensitive (80.8%), echogenic subendometrial lines and buds were the most specific (95.5%), and the globular uterus was the most accurate (80%) criteria (8). Similar to Kepkep et al.'s result, echogenic subendometrial lines and buds were analyzed as the most specific (95.5%) feature. According to our study, the sensitivity (57.7%) and accuracy (59%) of the globular uterus were found to be low. Although the groups in the study population were found to be similar in terms of leiomyoma, the predictivity of the globular uterus was found to be statistically significant. It was concluded that the globular uterus feature has an important place in the diagnosis of adenomyosis, even in the presence of leiomyoma. The results of our study are partially similar to those in the literature. The use of different sonography techniques and criteria in studies creates differences in the results.

In revised definitions of MUSA features of adenomyosis, researchers suggested that the echogenic subendometrial buds and lines feature may lead to diagnostic confusion between adenomyosis and malignancies in older and postmenopausal patients (5). In our study, gynecological malignancies were excluded from the study. Hyperechogenic islands and echogenic subendometrial buds and lines features were significant differences in the adenomyosis group. Echogenic subendometrial buds and lines and hyperechogenic islands had low sensitivity (15 and 17%, respectively). However, they had high specificity (95.5 and 94%, respectively), high NPV (81 and 94%, respectively), high accuracy (79 and 78%, respectively), and high positive LR (3.3 and 2.9, respectively). The predictivity of both criteria was found to be statistically significant in univariate regression analysis. The specificity, NPV, PPV, and accuracy of these direct features were remarkable.

It has been stated that the irregular junctional zone was weaker than other criteria in the revised definitions of MUSA features of adenomyosis (5). Contrary to this view, Tellum et al. reported that this feature reflects good discrimination ability (6). The common opinion in the literature is that junctional zone evaluation should be performed by expert gynecologists in 3D TVS (5, 6, 12). According to our analysis, the irregular junctional zone feature was a significant difference in the adenomyosis group. The irregular junctional zone had 32.5% sensitivity, 82.6% specificity, and 82.6% NPV. Its predictivity was observed to be statistically significant in univariate regression analysis. In our study, junctional zone evaluation was performed by an expert gynecologist on 3D TVS, as recommended in the current literature.

As stated in the revised definitions of MUSA features of the adenomyosis study, it is unclear which feature or features are required to diagnose adenomyosis (5). In our study, we determined the cutoff values for direct features >1 and indirect features >4. These values were not statistically significant. However, the total number of features was 1.5 times more significantly different in the adenomyosis group. Moreover, the cutoff value of total features >4 was statistically significant. Four combinations that provide this cutoff value were identified. However, the sensitivities of the combinations were found to be quite low. “0 direct + 5 indirect” and “1 direct + ≥ 4 indirect” were the weakest combinations among the others, with an accuracy of 78 and 77%, respectively. The combination with the highest specificity, PPV, NPV, and accuracy was “3 direct + ≥ 2 indirect” (100, 100, 95.1, and 80.6%, respectively). As mentioned in the revised definitions of MUSA features of the adenomyosis study, all three direct traits might not be present in the same uterus, and direct features are frequently modest and difficult to see. It could be simpler to identify indirect traits than direct ones (5). In this regard, the above combinations can be included in adenomyosis sonography practice.

Zannoni et al. aimed to determine the diagnostic accuracy of ultrasound features related to adenomyosis according to the MUSA statement and two additional markers (question mark sign and TVS uterine tenderness) (13). In the adenomyosis group, compared to the control group, the question mark sign was approximately 10 times higher and the uterine tenderness was ~2 times higher. It has been reported in the literature that the question mark sign may be a marker of adenomyosis, which is strongly associated with posterior deep infiltrating endometriosis (14, 15). In that study, the question mark was proven to be an independent marker of adenomyosis. The question mark sign also showed great specificity (96%) and PPV (83%) for 2D TVS features. The authors reported that these results suggest that the question mark sign may have a broader application in diagnosing adenomyosis than previously thought. It is known that there is a relationship between adenomyosis and pelvic pain, especially in patients with adenomyosis accompanied by endometriosis. The use of TVS as a dynamic examination can indicate whether the pain is due to gentle pressure and mobilization of the uterus. The sensitivity of uterine tenderness was found to be 67.3%, and the NPV was 81%.

Several studies have described the relationship between ultrasound features of adenomyosis and clinical outcomes (16–18), but MUSA descriptions of ultrasound features have been addressed in only one of them (18). It was reported that women with TVS features of adenomyosis had more severe menstrual pain than women without these features, and a positive correlation was reported between the number of ultrasound features and the severity of menstrual pain (18). The relationship between the presence of one or more direct or indirect MUSA features and clinical symptoms, as well as the relationship between the number and size of features and their location and symptoms, also needs to be further investigated. Since the reference standard is hysterectomy, it is difficult to perform clinically useful diagnostic accuracy studies in women with suspected adenomyosis who are not planned for surgery. Additionally, there is no common guideline regarding histopathological diagnostic criteria for adenomyosis. For this reason, there is no standard approach among pathologists (5).

Raimondo et al. evaluated the diagnostic performance of the deep learning (DL) machine for the detection of adenomyosis on uterine ultrasonographic images and compared it to intermediate ultrasound skilled trainees (19). The DL model achieved a low diagnostic performance for the detection of adenomyosis with an accuracy of 51%, lower than that of intermediate-skilled trainees. The sensitivity of the intermediate-skilled trainees was higher than that of DL as well. However, the DL model showed potential for excluding adenomyotic uteri, with higher specificity and NPV than those of intermediate-skilled trainees (19).

The robustness of our study was underscored by several key strengths. First, the utilization of an updated classification system, optimized and standardized through the revised definitions of MUSA features of adenomyosis, ensures a contemporary and consistent framework for analysis. The incorporation of 3D TVS for the examination of features adds a layer of sophistication to our methodology, enabling a more nuanced and detailed assessment. Conducting the study within a single tertiary center contributes to result homogeneity, minimizing potential external influences. The limitations of our study were its retrospective design, the heterogeneity of hysterectomy indications, and the inclusion of patients with multiple and large leiomyomas. Additionally, while interobserver agreement assessment was conducted, intraobserver agreement assessment was not performed.

In conclusion, this study shows that combinations with a total number of features >4 can be practically used in the evaluation of adenomyosis using the revised definitions of MUSA features. Prospective studies correlating ultrasound findings with standardized histopathological criteria and clinical findings will yield more accurate and precise results. Moreover, in the future, DL will be used more effectively in the diagnosis of adenomyosis.

## Data availability statement

The raw data supporting the conclusions of this article will be made available by the authors, without undue reservation.

## Ethics statement

The studies involving humans were approved by Dokuz Eylül Unvisersity Institutional Ethics Committee (File number: 7737-GOA, Registration number: 2023/02-13). The studies were conducted in accordance with the local legislation and institutional requirements. Written informed consent for participation was not required from the participants or the participants' legal guardians/next of kin in accordance with the national legislation and institutional requirements.

## Author contributions

OY: Conceptualization, Data curation, Formal analysis, Investigation, Methodology, Project administration, Resources, Software, Supervision, Validation, Visualization, Writing – original draft, Writing – review & editing, Funding acquisition. AA: Formal analysis, Writing – original draft, Data curation. MÖ: Data curation, Software, Writing – original draft. BE: Data curation, Writing – original draft. SK: Supervision, Validation, Writing – review & editing. EU: Data curation, Writing – original draft. MG: Project administration, Supervision, Visualization, Writing – review & editing.
